# Challenge of conducting a placebo-controlled randomized efficacy study for influenza vaccine in a season with low attack rate and a mismatched vaccine B strain: a concrete example

**DOI:** 10.1186/1471-2334-9-2

**Published:** 2009-01-17

**Authors:** Jiří Beran, Veronika Wertzova, Karel Honegr, Eva Kaliskova, Martina Havlickova, Jiří Havlik, Helena Jirincova, Pascale Van Belle, Varsha Jain, Bruce Innis, Jeanne-Marie Devaster

**Affiliations:** 1 The Vaccination and Travel Medicine Center, Hradec Kralove, Czech Republic; 2 Department of Infectious Diseases, University Hospital, Hradec Kralove, Czech Republic; 3 National Institute of Public Health, National Reference Laboratory for Influenza, Praha, Czech Republic; 4 GlaxoSmithKline Biologicals, Praha, Czech Republic; 5 GlaxoSmithKline Biologicals, Rixensart, Belgium; 6 GlaxoSmithKline Biologicals, King of Prussia, USA

## Abstract

**Background:**

Our aim was to determine the efficacy of a trivalent inactivated split virus influenza vaccine (TIV) against culture-confirmed influenza A and/or B in adults 18 to 64 years of age during the 2005/2006 season in the Czech Republic.

**Methods:**

6203 subjects were randomized to receive TIV (N = 4137) or placebo (N = 2066). The sample size was based on an assumed attack rate of 4% which provided 90% power to reject the hypothesis that vaccine efficacy (VE) was ≥ 45%. Cases of influenza like illness (defined as fever (oral temperature ≥37.8°C) plus cough and/or sore throat) were identified both by active (biweekly phone contact) and passive (self reporting) surveillance and nasal and throat swabs were collected from subjects for viral culture.

**Results:**

TIV was well tolerated and induced a good immune response. The 2005/2006 influenza season was exceptionally mild in the study area, as it was throughout Europe, and only 46 culture-confirmed cases were found in the study cohort (10 influenza A and 36 influenza B). Furthermore among the B isolates, 35 were identified as B/Hong Kong 330/2001-like (B/Victoria/2/87 lineage) which is antigenically unrelated to the vaccine B strain (B/Yamagata/16/88 lineage). The attack rate in the vaccine group (0.7%) was not statistically significantly different from the attack rate in the placebo group (0.9%).

**Conclusion:**

Due to the atypical nature of the influenza season during this study we were unable to assess TIV efficacy. This experience illustrates the challenge of conducting a prospective influenza vaccine efficacy trial during a single season when influenza attack rates and drift in circulating strains or B virus lineage match can be difficult to estimate in advance.

**Trial Registration:**

Clinical trial registery: NCT00197223.

## Background

Influenza is a highly contagious infectious disease resulting in acute respiratory illness in people of all ages. Annual epidemics occur worldwide and cause substantial morbidity and mortality [1,2]. Influenza poses a particular risk to the elderly and also to people suffering from conditions such as chronic heart or pulmonary disease. The causative agents are influenza A and influenza B viruses and the main virulence factors are the virus surface coat proteins hemagglutinin (HA) and neuraminidase (NA). There are several antigenic forms of HA and NA for influenza A which is classified into subtypes based on different combinations of these antigens [1,3,4]. Only a few of these influenza A subtypes have ever been associated with human disease and the subtypes currently in circulation in human hosts are H1N1 and H3N2 [5]. The influenza B virus currently belongs to two evolutionary lineages that are distinct at the genetic and antigenic levels and which are represented by B/Yamagata/16/88-like and B/Victoria/2/87-like viruses that have co-circulated in the population since the mid-1980s [5-8]. In order to evade the host immune system, the HA and NA proteins of both influenza A and influenza B viruses undergo continuous mutation and by this mean evade the host immune system. This is known as antigenic or genetic drift [1,5,9,10].

Influenza vaccination has been employed for many years as the primary tool to prevent influenza virus infection and its complications [2]. As recommended by the World Health Organization (WHO), vaccines are trivalent containing two influenza A strains (H1N1 and H3N2) and one influenza B strain [1]. However, to ensure efficacy against new drift variants the vaccine strains must be updated on an annual basis for both the Northern and the Southern hemisphere [11]. Based on epidemiology and phylogenetic analysis of HA and NA sequences of the circulating human strains detected though a global influenza surveillance network, WHO recommends the three strains that are anticipated to become dominant during the next influenza season [11]. Although in most years the recommendations accurately predict a close antigenic match between the vaccine and circulating strains, occasionally a predominant circulating strain turns out to be antigenically different from the corresponding vaccine strain. As two influenza B virus lineages co-circulate, the current recommendation to include only one lineage in each year's TIV poses a particular risk for a mismatch. Vaccine strain mismatch can have a negative impact on vaccine efficacy [9,10,12,13].

In this paper we describe an efficacy study conducted with a trivalent inactivated split-virus influenza vaccine (TIV) manufactured by GlaxoSmithKline Biologicals which has been available since 1987 and is now used in over 100 countries [4,14,15]. As for other inactivated influenza vaccines, the ability of TIV to induce HA antibody levels (as measured in hemagglutination-inhibition (HAI) assay) that meet regulatory authority criteria has been accepted as a surrogate marker for efficacy [4,14,15]. Recently, this TIV vaccine was approved under the United States' accelerated approval regulation [4,15]. To fulfill the requirements of this regulation, post-approval demonstration of clinical benefit in an adequate and well-controlled clinical trial is required.

We designed a placebo-controlled randomized efficacy study with a primary clinical endpoint based upon rates of culture-confirmed influenza A and/or B disease in a target population of healthy adults with a broad age range of 18 to 64 years. All influenza vaccine efficacy studies are subject to the unpredictable nature of the intensity of the influenza season in which they are conducted as well as the extent of the antigenic match between the circulating and vaccine strains [16]. As illustrated by the outcome of this present study, these factors can have a substantial negative impact on the assessment of vaccine efficacy.

## Methods

### Study design and participants

This was a randomized, double-blind, placebo-controlled study with two groups conducted in the Czech Republic in the 2005–2006 influenza season (Clinical trial registery: NCT00197223). The protocol and study documents were approved by the University Hospital Hradec Kralové Ethics Committee (Sokolska, 581; 50005 Hradec Kralové, Czech Republic). Our primary objective was to evaluate the efficacy of TIV (2005/2006 Northern Hemisphere formulation) compared with placebo in the prevention of culture-confirmed influenza A and/or B disease in adults. A secondary objective was to assess the efficacy of TIV to prevent influenza like illness (ILI) during the influenza season (which was defined as starting the first week with two culture-confirmed cases in the study area, and ending the last week with one culture-confirmed case in the study area). Other secondary objectives were the assessment of the safety and also, in a subset of subjects, the assessment of vaccine reactogenicity and immunogenicity.

Eligible participants were self-referred clinically healthy male or female volunteers aged between 18 and 64 years at the time of vaccination who provided written informed consent. A randomisation list was generated by the sponsor by SAS program and used to number the vaccine and placebo treatments (which were indistinguishable in appearance). A randomization blocking scheme (2:1) was employed to ensure that balance between treatments was maintained. The randomization algorithm used a minimization procedure that accounted for prior influenza vaccination, vaccine lots, and agreement to participate in the immunogenicity/reactogenicity subset. Blinding to treatment assignment was maintained until study analysis.

### Vaccinations and blood sampling

Volunteers were randomized to receive one dose of TIV (*Fluarix*™, a trade mark of GlaxoSmithKline group of companies (GSK)) or placebo at the first study visit (study Day 0) by intramuscular injection into the non-dominant deltoid muscle. Each 0.5-ml dose of TIV contained 15 μg each of the hemagglutinin antigens of A/New Caledonia/20/99 (H1N1) IVR-116 virus as an A/New Caledonia/20/99-like strain, A/New York/55/2004 (H3N2) X-157 virus as an A/California/7/2004-like strain and B/Jiangsu/10/2003 virus as a B/Shanghai/361/2002-like strain. Placebo consisted of saline for injection.

Blood samples for the assessment of immunogenicity were obtained from the planned randomly selected subset of volunteers just prior to vaccination and at 21 days following vaccination.

### Case definitions

Culture-confirmed influenza (primary endpoint) was defined as an episode of influenza like illness (ILI) occurring after the administration of the study vaccine for which a nasal and throat swab specimen yields influenza virus A and/or B in cell culture.

ILI (secondary endpoint) was defined as fever (oral temperature ≥ 37.8°C) plus cough and/or sore throat [17]. An ILI episode was defined as the period from the first day of ILI symptoms until the last day of ILI symptoms. A new episode was taken into account only after the complete resolution of the previous one. Between two ILI episodes, there had to be at least 7 days free of any symptoms.

### Follow up of subjects

From the day of vaccination all subjects were instructed to report any ILI symptoms to the investigator via a toll free line (passive surveillance for ILI). Active surveillance for ILI began 2 weeks after the administration of the study vaccine until study end (April 2006). This involved biweekly telephone contact of the subjects to remind them to notify the investigator at the onset of ILI symptoms, to identify ILI episodes which may have been unreported, and to collect information on the occurrence of medical visits and possible serious adverse events.

For each case of suspected ILI, a study nurse visited the subject, (as far as possible on the same day) and collected a nasal and throat swab sample (comprised of a swab of both nasal cavities and a second swab of the throat) for culture. Subjects were each provided with a calibrated thermometer to measure temperature and a diary card to record temperatures and symptoms during the influenza like illness episode. All samples were to be obtained, if possible, before antimicrobial/influenza antiviral therapy was started.

A randomly selected subset of subjects were assessed for reactogenicity using diary cards to record the presence and intensity of injection site solicited adverse events (pain, redness and swelling) and general solicited adverse events (fatigue, fever, headache, muscle aches, shivering and joint pain) experienced during the first 4 days after vaccination. The diameters of any injection site redness or swelling and daily body temperature were recorded. The intensities of other adverse events were recorded according to a standard 0 to 3 grade scale: "absent", "easily tolerated", "interferes with normal activity" and "prevents normal activity". Data were also collected on the occurrence and intensity of any spontaneous unsolicited signs or symptoms occurring within 21 days following vaccination. These diary cards were reviewed during the blood sampling visit at Day 21. Collection of data on serious adverse events (SAEs) began at the receipt of vaccine/placebo and continued until the end of the study.

At the end of the study the investigator made a study conclusion contact with all subjects.

### Laboratory assays

Serum samples were stored at -20°C until blinded analyses were conducted at GSK Biologicals SSW Dresden, Germany. All samples were tested in a validated micro-titer hemagglutination-inhibition test using chicken red blood cells with the three virus strains present in the TIV used as antigens as previously described [14]. The serum titer was expressed as the reciprocal of the highest dilution that showed complete inhibition of hemagglutination.

Nasal and throat swab samples were stored at 2–8°C and transferred within 24 hours to the National Reference Laboratory for Influenza (NRL, Prague, Czech Republic) for conventional influenza virus culture in Madin Darby Canine Kidney (MDCK) cells as described elsewhere [18,19]. Cell culture supernatants were tested for the presence of influenza virus by hemagglutination assay with turkey and guinea pig erythrocytes [18,19]. Hemagglutination inhibition was used to identify virus type, subtype and drift variant [18,19]. Cell culture supernatants were also tested by a direct immunoperoxidase assay using anti-influenza A and anti-influenza B nucleoprotein antibodies [20].

### Statistical Analysis

The sample size for the primary endpoint was estimated on the basis of an assumed influenza attack rate of 4% of culture-confirmed influenza disease (type A and/or B) among placebo recipients, a vaccine efficacy (VE) of 70%, and 90% power to detect vaccine efficacy with a lower limit of the 95% CI for efficacy >45% with alpha = 0.025 (1-sided). A sample size of 5591 evaluable subjects was required to allow the rejection of the null hypothesis that the vaccine efficacy against culture-confirmed influenza illness was ≤ 45%. To account for an assumed dropout rate of 10%, we planned to enroll a minimum of 6213 subjects. According to the planned 2:1 treatment allocation approximately 4142 and 2071 subjects were to receive TIV and placebo respectively. Based on the assumptions of attack rate and vaccine efficacy a total of 119 culture-confirmed influenza illness episodes were anticipated with approximately 75 episodes in the placebo group.

**Figure 1:**
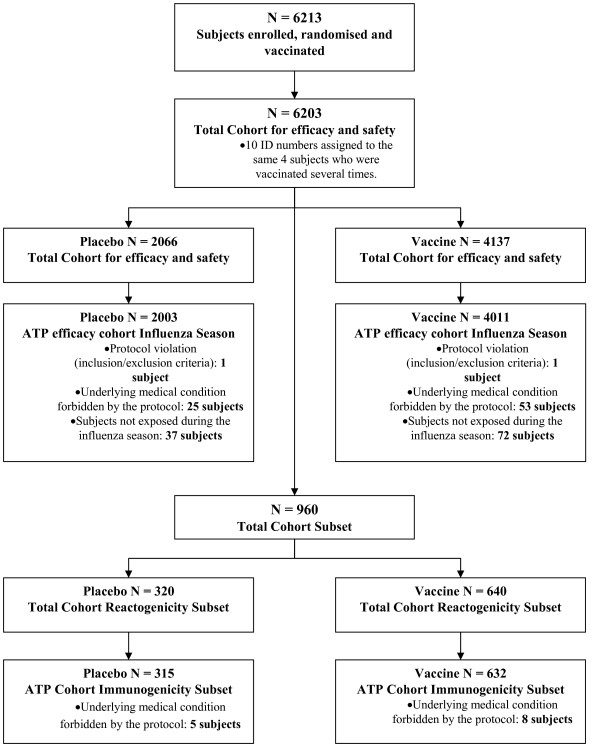
**Flow of participants through the clinical trial**.

All data analyses were conducted according to a pre-specified plan prepared by GSK and submitted to the United States Center for Biologics Evaluation and Research (CBER). The vaccine efficacy against culture-confirmed influenza A or B was defined as 1 minus the relative risk of culture-confirmed influenza among the TIV group versus the placebo group and calculated using the classical formula of Greenwood and Yule [21] with the 95% exact confidence intervals (CI). The attack rate was the number of subjects experiencing at least one episode of culture-confirmed influenza A or B within the observation period divided by the total number of subjects in the group. Analysis of vaccine efficacy against culture-confirmed influenza A or B was performed on the total cohort for efficacy (Figure 1) while analysis of vaccine efficacy against ILI during the influenza season was performed on the according to protocol (ATP) cohort for efficacy in the influenza season (Figure 1).

## Results

### Baseline characteristic

The total cohort for efficacy and safety (N = 6203 subjects) included 4137 subjects vaccinated with TIV and 2066 subjects vaccinated with placebo (Figure 1). The demographic profiles of the TIV and placebo groups in this cohort were comparable with respect to mean age (35 ± 13 years in both groups), gender (55.3% and 54.2% female in the TIV and placebo groups respectively) and racial distribution (99.8% white Caucasian in both groups). In both groups the majority of subjects (91.5% and 91.7%) had no history of influenza vaccination within the last 3 influenza seasons prior to enrolment.

### Efficacy

During the study surveillance period (September 2005 to April 2006), 814 episodes of ILI were evaluated with the peak incidence occurring between November 2005 and March 2006 (110–125 cases per month). Swab specimens were obtained from all cases within 5 days of the onset of ILI with the majority (742/814, 91%) collected by Day 2. There were 46 culture-confirmed cases, 10 positive for influenza A and 36 for influenza B (Table 1) which occurred between January and April 2006. Seven of the ten influenza A isolates were identified by hemagglutination inhibition titration and characterised as being antigenically related to the H1N1 or H3N2 vaccine strains (Table 1). Of the 36 influenza B isolates, 35 were identified by hemagglutination inhibition titration and characterized as B/Hong Kong 330/2001-like (from the B/Victoria/2/87 lineage) which is antigenically distinct to the B strain from the B/Yamagata/16/88 lineage contained in the vaccine. No mixed influenza infections were identified.

**Table 1:** Culture confirmed influenza episodes identified by immunoperoxidase staining or by hemagglutination inhibition titration

Total Number of Influenza A or B isolates	Number identified by immunoperoxidase typing only	Number identified by hemagglutination inhibition for A/H1, A/H3 and B lineage
**10 Influenza A isolates**	**3**	**5 **H3 A/California/7/2004-like*
		
		**2 **H1 A/New Caledonia/20/1999* and A/Czech Republic/109/2005*

**36 Influenza B isolates**	**1**	**35** B/Hong Kong 330/01-like**

* Antigenically related to the H1N1 or H3N2 vaccine strains

** Not antigenically related to the B strain from the Yamagata lineage contained in the vaccine

As indicated in Table 2 the attack rate for culture-confirmed influenza A or B in the placebo group was 0.9% which was well below the assumed attack rate of 4% on which the sample size was based to offer 90% power to reject the null hypothesis that VE was ≤ 45%. The attack rate in the vaccine group (0.7%) was not statistically significantly different so the null hypothesis could not be rejected and the efficacy of TIV against culture-confirmed influenza A or B could not be demonstrated (estimate of efficacy of 22.3% with 95% CI of -49.1% to 58.5%). The attack rate for culture-confirmed influenza A in the placebo group was very low at only 0.2%. For culture-confirmed influenza B (Table 2), the attack rates were 0.7% in the placebo group and 0.5% in the vaccine group and the lower limit of confidence for the estimate of 21.5% efficacy was again a negative value (95% CI -65.9% to 61.6). Similarly the attack rates for clinical ILI episodes during the influenza season were not statistically different between the vaccine (6%) and placebo (5.6%) groups and so efficacy against clinical ILI episodes could also not be demonstrated (Table 3).

**Table 2:** Attack rates and TIV efficacy for culture confirmed influenza in adults (total cohort for efficacy)

Type of culture confirmed	Trivalent inactivated split virus influenza vaccine N = 4137		Placebo N = 2066		Vaccine efficacy (1-RR)			
	
	Number of cases	% Attack rate	Number of cases	% Attack rate	%	95% CI†		P value †
							
						LL	UL	
**Any**	28	0.7	18	0.9*	**22.3**	-49.1	58.5	0.433

**Influenza A**	6	0.1	4	0.2	**25.1**	-260.9	82.2	0.740

**Influenza B**	22	0.5	14	0.7	**21.5**	-65.9	61.6	0.481

* Sample size calculation was based on expected attack rate of at least 4% in the placebo group

† Two-sided Fisher Exact test, the 95% confidence intervals for vaccine efficacy was calculated using a conditional exact method.

**Table 3:** Attack rates and TIV efficacy for ILI during the influenza season* (ATP cohort for efficacy)

Trivalent inactivated split virus influenza vaccine N = 4011			Placebo N = 2003			Vaccine efficacy			
						
						%	95% CI†		p value †
			
Number of events	Number of subjects reporting at least one event	% Attack rate	Number of events	Number of subjects reporting at least one event	% Attack rate		LL	UL	
254	240	6.0	120	113	5.6	-6.1	-33.8	15.5	0.642

* Influenza season starts the first week with two culture confirmed cases in the area, and closes at the end of the last week with one culture confirmed case in the areas.

† Two-sided Fisher Exact test, the 95% confidence intervals for vaccine efficacy was calculated using a conditional exact method.

### Safety and reactogenicity

The percentage of subjects reporting an SAE was the same (2.3%) in each group. No SAEs were reported as being related to vaccination.

**Figure 2:**
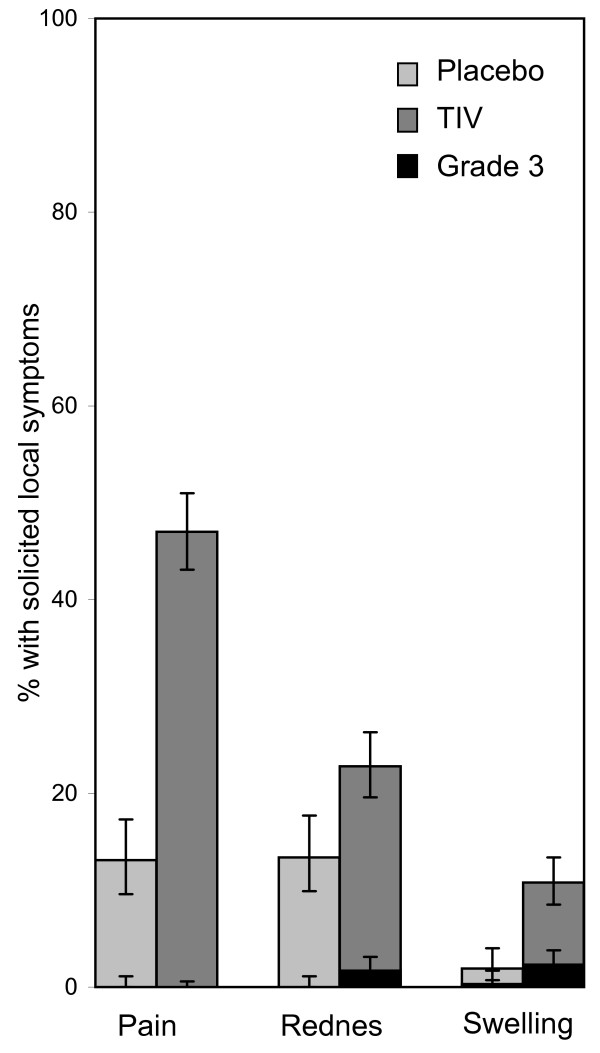
**Incidence of solicited injection site symptoms during the first four days following vaccination (Total cohort subset for reactogenicity; trivalent inactivated split virus influenza (TIV) N = 640, Placebo N = 320)**.

**Figure 3:**
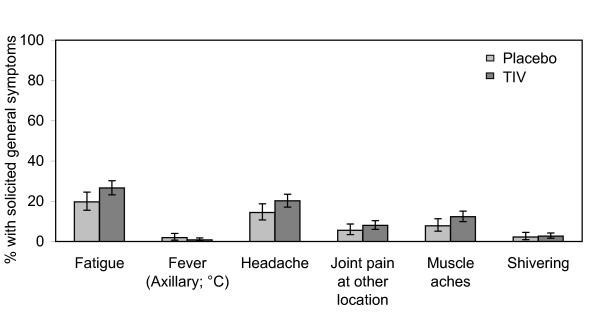
**Incidence of solicited general symptoms during the first four days following vaccination (Total cohort subset for reactogenicity; trivalent inactivated split virus influenza (TIV) N = 640, Placebo N = 320)**.

Vaccination with TIV was associated with a higher incidence of solicited injection site reactions, in particular pain at the injection site (Figure 2). The majority of these reactions in TIV recipients were mild to moderate in intensity. No events were classified as preventing daily activities and the incidences of local redness and swelling reactions above 50 mm diameter were low (1.7% and 2.3% respectively). All resolved without sequelae. Rates of solicited general symptoms also tended to be higher in the TIV group (Figure 3). Fatigue was the most common solicited general symptom reported as related to vaccination in both groups (TIV 23.4% and placebo 15.9%). The majority of reactions in TIV recipients were mild to moderate in intensity, there were no reports of fever >39°C and the highest incidence of symptoms preventing daily activities and reported as related to vaccination was 0.5% (fatigue and headache). Rates of spontaneously reported symptoms were similar in both groups both overall (TIV 11.1% and placebo 10%) and reported as related to vaccination (TIV 1.4% and placebo 1.6%).

### Immunogenicity

Table 4 demonstrates that TIV elicited an antibody response (as assessed by hemagglutination-inhibition) against all three vaccine strains which exceeded the required Committee for Medicinal Products for Human Use (CHMP) [22] and CBER [23] criteria for seroconversion and seroprotection rates.

**Table 4:** Hemagglutination-inhibition antibody response at 21 days following vaccination (ATP cohort subset for immunogenicity)

			Influenza A (H1N1) *A/New Caledonia/20/99 (H1N1) IVR-116*		Influenza A (H3N2) *A/New York/55/2004 (H3N2) X-157*		Influenza B *B/Jiangsu/10/2003 virus*	
	**CHMP** **acceptance** criteria**	**CBER** acceptance criteria***	***TIV*** **N = 632**	**Placebo** **N = 315**	***TIV*** **N = 632**	**Placebo** **N = 315**	***TIV*** **N = 632**	**Placebo** **N = 315**

**Geometric Mean Titer (GMT)**	No Standard	No Standard	730.5 [648.1–823.3]	11.6 [10.2–13.3]	131.7 [119.9–144.6]	13.5 [11.9–15.3]	191.1 [175.7–207.9]	17.9 [15.7–20.3]

% Sero-conversion rate *	% > 40%	LL of 95% CI > 40%	89.2 [86.6–91.5]	0.0 [0.0–1.2]	77.2 [73.7–80.4]	0.3 [0.0–1.8]	82.9 [79.7–85.8]	0.0 [0.0–1.2]

**% Sero-protection** **(titer ≥ 40)**	% > 70%	LL of 95% CI > 70%	97.8 [96.31–98.78]	21.9 [17.46–26.88]	88.1 [85.35–90.55]	22.5 [18.04–27.56]	95.9 [94.03–97.30]	30.2 [25.14–35.56]

* Seroconversion rate for Hemagglutination-inhibition antibody response is defined as the percentage of vaccinees who have a prevaccination titer < 1:10 and a post-vaccination titer ≥ 1:40, or who have a prevaccination titer ≥ 1:10 and at least a fourfold increase in post-vaccination titer

## Discussion

This paper describes the outcome of a placebo-controlled randomized efficacy study with a trivalent inactivated split virus influenza vaccine (TIV) in healthy adults aged 18 to 64 years. The immunogenicity of TIV was consistent with previous studies in adults [4,15] and seroprotection rates exceeded regulatory authority criteria. Despite this, the vaccine efficacy was not confirmed.

Influenza vaccine efficacy in a particular region depends on the nature of the influenza season. This can vary from year to year and as it cannot be predicted in advance, poses a particular challenge for the conduct of a prospective efficacy study. The optimal circumstances in which to evaluate the efficacy of influenza vaccines are i) high viral circulation (i.e. high attack rate) and ii) when the vaccine strains are antigenically-matched to the circulating virus types [16,24]. The 2005–2006 influenza season in the Czech Republic was exceptional in that both of these factors turned out to be suboptimal for the conduct of an efficacy study.

The first issue faced by the study was the very low attack rate. The study was powered based on the assumption that the attack rate of culture-confirmed influenza in the placebo group would be 4% while the actual attack rate during the study was 0.9%. Literature data are limited on the attack rates of culture-confirmed influenza cases in an unvaccinated adult population and serology has been very often used alone or combined as a diagnostic tool to confirm the influenza virus etiology of a clinical case. The attack rate of 4% was hence selected as a conservative estimate based upon data from randomized placebo influenza vaccine studies in healthy adults with laboratory (by serology or a mixture of serology and culture) confirmed endpoints reviewed in the Cochrane data base [3]. These indicated an attack rate range of 6% to 16% in the placebo groups. More recently, prospective placebo-controlled trials have reported rates of culture-confirmed cases in the adult setting [25,26] as well as in children [27]. These studies clearly confirmed the high year-to-year variability of documented influenza cases.

The low number of culture-confirmed influenza cases found during our study was unlikely to be due to inadequate monitoring of prospective cases, as active surveillance for ILI involving contact of subjects by phone on a biweekly basis was employed. Also, all nasal and throat swab specimens were transported to the laboratory with an adequate cold chain within 24 hours to avoid false culture negatives due to loss of virus viability. Furthermore, the low attack rate observed in the study area in 2005–2006 was confirmed by the European Influenza Surveillance Scheme (EISS) [28]. Specific data for the Czech Republic demonstrate that the weekly morbidity rates for both acute respiratory infections [29] and for ILI [30] were lower in the 2005/2006 influenza season compared to both the previous 2004/2005 season and also the following 2006/2007 season. The EISS also reported that the circulation of influenza B virus in the winter 2005–2006 was exceptional compared with European data accumulated during the last decade, as it was the only season in which influenza B viruses were dominant [28]. It is possible that this may have contributed to the low attack rate of clinical illness as influenza B is known to cause milder disease than influenza A [28,31].

The significant impact that a lower attack rate can have on a clinical trial to evaluate influenza vaccine efficacy has previously been illustrated in a clinical trial conducted by Hoberman and colleagues in young children over two consecutive influenza seasons in Pittsburg [27]. In the first season (1999/2000) when the attack rate for culture-confirmed influenza was 15.9% in the placebo group, vaccine efficacy was estimated to be 65% (95% CI 34% to 82%) while in the second season (2000/2001) when the attack rate for culture-confirmed influenza was 3.3% in the placebo group, vaccine efficacy was estimated to be -7% (95% CI -247% to 67%). A similar finding was recently reported in a study conducted over 2 years in an adult population in Michigan [25,26]. The attack rate of culture-confirmed influenza in the placebo group was reported to be 5.8%, whereas in the second year, a rate of 1.8% was found. The VE estimates for the inactivated influenza vaccine were found to be 77% (37% to 92%) in year 1 and 23% (-153% to 73%) in year 2. The latter result appears to be similar to the outcome of our study in adults where with an attack rate in the placebo group of 0.9% for influenza A and/or B cases and 0.2% for influenza A cases, the respective estimate of efficacies were 22.3% (95% CI -49.1% to 58.5%) and 25.1% (95% CI of -260.9% to 82.2%).

These data indicate that the possibility of a lower than predicted attack rate needs to be factored into the design of influenza vaccine efficacy studies. Apart from increasing the sample size or allowing for an extension of the study over more than one influenza season, another factor to be considered is the clinical case definition used. There is no universally accepted clinical case definition of influenza [32,33]. We employed a definition from the Centers for Disease Control and Prevention (CDC) US based on the presence of fever plus either cough or sore throat, [17]. This combination of symptoms is recognized as having a high positive predictive value for influenza but is not very sensitive [34]. For seasons with low attack rates it may be preferable to improve case capture by using less restrictive clinical criteria particularly when culture confirmation is being employed to confer specificity. The use of reverse polymerase chain reaction (RT-PCR) assays techniques, which have been shown to be more sensitive than culture, may also improve detection of influenza virus in nasal/throat specimens [35,36]. Serological analysis of acute and convalescent sera, which is also often used to confirm influenza cases, would however have specific limitations in a vaccine efficacy study. In the vaccine group, the presence of vaccine-induced antibodies could mask differences between paired sera in levels of antibodies induced by infection, thereby introducing a bias s in favor of the study vaccine.

The low attack rates encountered in the adult population during some influenza seasons may raise the question about the need for adult vaccination. It should be kept in mind that currently the main target populations for adult vaccination (apart from the elderly persons, which are outside the scope of this study) are adults who have medical conditions that place them at risk for complications from influenza and also, in order to limit influenza virus transmission to those at risk in all age groups, health-care personnel or caregivers [2]. As the attack rate for any coming influenza season is unpredictable at the time of annual influenza vaccination, it is not feasible to modify vaccination routines and put susceptible people at risk.

The second issue faced by the study was a mismatch between the dominant circulating influenza strain and the vaccine strains. The majority of culture-confirmed cases detected in our study were caused by an influenza B strain that was of a different lineage to the vaccine influenza B component which was from the B/Yamagata/16/88-lineage. This mismatch was confirmed by the EISS who reported that 90% of the characterized influenza B viruses in Europe during the influenza season 2005–2006 were similar to the B/Victoria/2/87 lineage of influenza B viruses [28]. As a consequence, the World Health Organization (WHO) substituted a virus from the B/Victoria/2/87 lineage in the 2006–2007 vaccine recommendations [37].

The co-circulation of these two antigenically distinct B lineages which form distinctly divergent genetic groups based on their hemagglutinin genes where there are some 27 amino acid differences [8] has raised the question of whether they should both be represented in the annual influenza vaccine formulation [38]. To date, this option has not been considered due to the constraints it would impose on an already limited influenza antigen manufacturing capacity. Also, although there is no hemagglutinin cross reactivity between the two B lineages in ferrets [7] or unprimed children [39], serological evidence from WHO [37] and another recent study [40] indicates that vaccine B/Yamagata/16/88-lineage components may provide reduced protection against the B/Victoria/2/87 lineage. Furthermore, reassortment between the two influenza B lineages has led to practically all B/Victoria-lineage viruses containing a B/Yamagata-lineage neuraminidase which may also provide some cross protection [13,38]. Other workers have managed to demonstrate vaccine efficacy for influenza B despite mismatch between vaccine and circulating strains [13,25]. In the Michigan study, Ohmit et. al demonstrated efficacy against influenza B during a mismatch of B strains in the 2004/2005 season [25]. However, the attack rate for culture-confirmed influenza in the placebo group in that study was considerably higher (5.8%) than in the study reported here and also a greater proportion of the influenza B isolates did match the vaccine strain (7 out of 18).

Although the exceptional nature of the influenza season in which this study was conducted did not allow us to establish the efficacy of TIV it is likely that, as demonstrated by Ohmit et al for another inactivated vaccine [25], TIV efficacy could be demonstrated under more typical influenza season conditions.

## Conclusion

Due to the exceptionally low attack rate during the 2005/2006 season in the Czech Republic and the predominance of a circulating B strain of a different lineage to the vaccine strain, our study was unable to demonstrate the efficacy of TIV in healthy adults. This experience illustrates the challenge of conducting an influenza vaccine efficacy trial during one season when variations in disease attack rates and circulating strains cannot be predicted in advance.

## Competing interests

The following authors are employed by the GlaxoSmithKline Group of Companies:

Jeanne-Marie Devaster, Bruce L Innis, Varsha Jain, Eva Kaliskova, Pascale Van Belle

The following authors hold stock options in GlaxoSmithKline:

Jeanne-Marie Devaster, Bruce L Innis, Varsha Jain

The following authors received reimbursements or fees or funding from GlaxoSmithKline: Jiří Beran, Martina Havlickova, Jiří Havlík, Helena Jirincova

The following authors have no conflict of interest: Karel Honegr, Veronika Wertzova

## Authors' contributions

All authors participated in the design, implementation, analysis and interpretation of the study. JB and JMD were involved in all phases of the study. JB was the co-ordinating and principal investigator. VW and KH led the clinical team respectively at the Vaccination and Travel Medicine Center and at the Department of Infectious Diseases assisted by JH. MH and HJ managed the team responsible for handling and processing of the ILI samples. BI and JMD led the clinical team at GSK Biologicals assisted by VJ and EK. PVB conducted the data analysis. All authors read and approved the final manuscript.

## Pre-publication history

The pre-publication history for this paper can be accessed here:

http://www.biomedcentral.com/1471-2334/9/2/prepub
